# Analysis of Composition, Structure, and Driving Factors of Root-Associated Endophytic Bacterial Communities of the Chinese Medicinal Herb *Glycyrrhiza*

**DOI:** 10.3390/biology14070856

**Published:** 2025-07-15

**Authors:** Zhilin Zhang, Aifang Ma, Tao Zhang, Li Zhuang, Hanli Dang

**Affiliations:** College of Life Sciences, Shihezi University, Shihezi City 832003, China; 18893536467@163.com (Z.Z.); 19119904935@163.com (A.M.); hamai_b@163.com (T.Z.); 18297081587@163.com (L.Z.)

**Keywords:** endophytic bacterial, medicinal plant species, plant nutrition, root depth, secondary metabolites, soil

## Abstract

The role of endophytic bacteria in the interaction between medicinal plants and microorganisms, secondary metabolite accumulation, plant nutrient changes, as well as their interactions with microbial communities, needs to be investigated in medicinal plants. In this study, 16S rRNA genes of endophytic bacterial communities in the root systems of three medicinal licorice species at different root depths (0–20, 20–40, and 40–60 cm) were sequenced using high-throughput sequencing technology, and their relationships with plant and soil factors were investigated. Our study indicated that the influence of *Glycyrrhiza* species on the structure of endophytic bacterial communities is significantly greater than that of root depth, and there are significant differences in the structure of endophytic bacterial communities at different sampling sites. At the phylum level, *Proteobacteria* and *Actinobacteria* are the dominant phylum. Functional gene prediction shows that functional genes related to metabolism dominate the endogenous bacterial community. Plant factors and soil physicochemical properties are important environmental drivers affecting the distribution of endophytic bacterial communities. This study will give new information on plant–soil–endophyte interactions and open up new possibilities for medicinal licorice development and use.

## Simple Summary

This study explores plant–soil–endophyte interactions in three licorice species, offering new insights for medicinal licorice development. It found significant differences in plant nutrients, secondary metabolites, and soil properties, influencing endophytic bacterial diversity. Licorice species had a greater impact on bacterial communities than root depth. The dominant bacterial phylum was *Proteobacteria*, followed by *Actinomycetes*. Functional genes related to metabolism were predominant. Environmental factors, particularly plant-related (37.02%) and soil-related (31.45%), drove bacterial distribution. pH and PC were identified as key factors affecting bacterial diversity and richness, suggesting that plant factors primarily influence bacterial community composition.

## 1. Introduction

*Glycyrrhiza* is a perennial herbaceous leguminous plant that can thrive in arid desert and semi-desert areas and has become one of the key resource plants in this ecological environment because of its resistance to cold, drought, salt and alkali, sand, heat, and light [1,2]. *Glycyrrhiza* is a significant medicinal plant, and the Chinese Pharmacopoeia listed the dried roots and rhizomes of *Glycyrrhiza uralensis*, *Glycyrrhiza glabra*, and *Glycyrrhiza inflata* as part of traditional Chinese herbal medicine [3]. They have been reported to contain a variety of active ingredients, including triterpene saponins and flavonoids [4]. Glycyrrhizic acid (GIA) and liquiritin (LI), a triterpene saponin and flavonoid, respectively [5], have an essential role in clinical medicine as anti-inflammatory, antiviral, immunomodulatory, anti-oxidation, and anti-bacterial agents [6,7]. Moreover, *Glycyrrhiza* is often utilized as a sweetener in the food service because of the presence of GIA, which possesses higher sweetness compared to sucrose [8,9]. The growth and biological characteristics of medicinal licorice plants, particularly their contents of active ingredients, have gradually become a focus of research in the medicinal plant field because of their outstanding medicinal and commercial importance.

Endophytic communities are an important part of the plant micro-ecosystem that reside in the intercellular space of all plants and are not associated with plant diseases or any significant morphological changes [10]. Endophytes live in a close symbiotic relationship with their host plants, which provide nutrients and shelter against most abiotic stresses [11]. Endophytic symbiosis provides plants with a variety of advantages, including increased plant hormones (indole-3-acetic acid, indole-3-acetonitrile, and cytokinins) to promote host plant growth [12], the production of different bioactive compounds (flavonoids and other phenolic antioxidants) to improve host plant resistance to biotic and abiotic stresses [13], and the promotion of secondary metabolite (paclitaxel and podophyllotoxin) accumulation [14]. However, to the best of our knowledge, there is no information on the comprehensive assessment of endophytic bacterial communities associated with medicinal licorice roots and their association with plant nutrients, secondary metabolites, and ecological environments.

During plant growth, plants select and enrich microorganisms from the soil to root-related compartments (e.g., rhizosphere and inner layer), each of which possesses a highly specific microbiome, which is influenced by plant genotypes [15,16]. Several studies have reported the use of Arabidopsis thaliana as a model plant for root-associated dominant or core bacteria [17] that is critical for understanding the assembly and stability of the root-associated microbiome. Different species of licorice plants may be accompanied by different rhizosphere-related and specifically enriched bacterial communities. However, few studies have focused on the specific effects of licorice species on the composition and structure of root-associated bacterial communities.

Many factors simultaneously influence the temporal dynamics of microbial communities in the natural environment. Therefore, determining the relative contribution of individual factors to the overall microbial succession process is quite difficult [18]. For the root-associated microbiome, the host plant’s genetic background, nutritional level, and ecological habitat (e.g., temperature, humidity, and light) are considered pressure-selective factors [19]. In this context, investigating the impact of plant nutritional status and soil characteristics on the growth and production of secondary metabolites in licorice plants can help anticipate how secondary metabolites and root-related bacterial populations respond to soil characteristics in different species of licorice plants. Therefore, understanding the effects of changes in plant nutrition and soil conditions on endophytic communities can have a significant impact on the growth of host plants and the optimization of licorice planting strategies.

Extensive characterization of endophytic bacterial communities associated with medicinal plants can help us better understand plant–-endophytic interactions, biological activity, and ecological roles [20,21], all of which can help us improve medicinal plant output and quality. Traditional research approaches, on the other hand, rely on culture identification to investigate the composition and biodiversity of endophytic bacteria. This in turn limits our understanding of the association between endophytic bacteria and plants. To enhance our understanding of the diversity of microbial populations in ecosystems, we need to use more powerful and accurate high-throughput sequencing (HTS), a method different from conventional culture, to detect the constitute and structure of endophytic bacterial communities in medicinal plants. In this research, root and soil samples of three medicinal licorice species were collected from three root depths, and HTS technology was used to explore the diversity and structure of endophytic bacterial communities within licorice roots. The study objectives are (1) to determine the differences in nutritional composition and the concentration of root secondary metabolite among three licorice species, as well as their relationship to environmental habitat (soil factors); (2) to explore the response of root-associated endophytic bacterial community composition and diversity to species and root depths of licorice; and (3) to investigate the role of plant and soil factors in the formation of endophytic bacterial communities and identify the role of endophytic bacterial communities in medicinal licorice.

## 2. Results

### 2.1. Plant and Soil Properties

Variance analysis showed that the plant nutrients and root secondary metabolites of three species of licorice were significantly affected by the species. As Table 1, the root water content (RWC) of D was significantly higher than that of G and W (*p* < 0.05). The plant organic carbon (PC) and Glycyrrhizic acid (GIA) content in G were significantly higher than those in D (*p* < 0.05). The contents of plant nitrogen (PN) and Liquiritin (LI) in W were significantly higher than those in G and D (*p* < 0.05). There were no significant differences in the contents of plant phosphorus (PP), potassium (PK), and root total flavonoids (GTF) among the three species of licorice (*p* > 0.05).

While there were no main differences in soil total nitrogen (STN) or PH among the three species of licorice (*p* > 0.05), there were main differences in the physical and chemical properties of other soil (SOM, STP, STK, SNN, SAN, SAP, SAK, TS, and SWC) among the three species of licorice (*p* < 0.05) (Table 1), even though the trend of difference was inconsistent.

**Figure 1:**
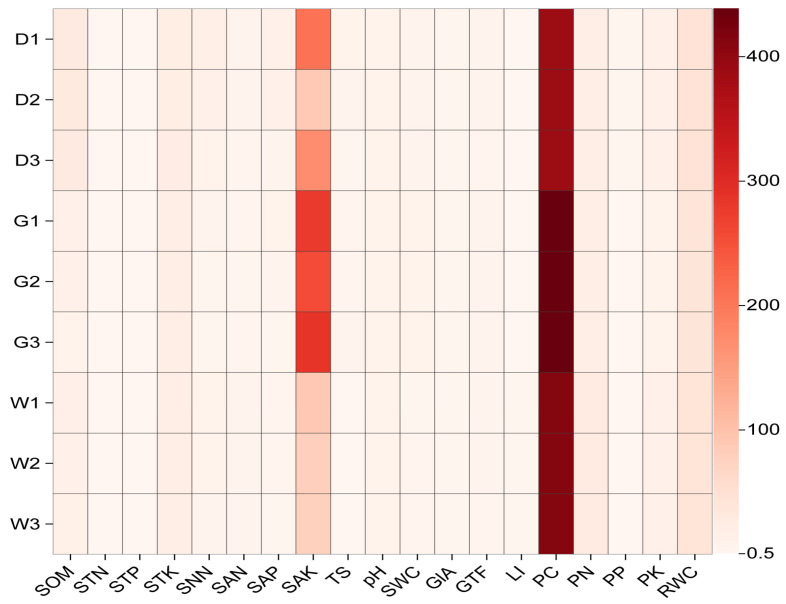
Heatmap of Spearman correlation analysis between soil physicochemical properties, plant nutrients, root secondary metabolites and root depth and species. Description: Corrcoef are the Spearman correlation coefficients. The horizontal coordinate is consistent with Table 1. The ordinate is the group name (D, G, and W: *Glycyrrhiza inflata*, *Glycyrrhiza glabra*, and *Glycyrrhiza uralensis*, respectively; 1, 2, and 3: root depth 0–20 cm, 20–40 cm, and 40–60 cm, respectively).

As shown in Figure 1, the heatmap shows a positive correlation between soil physicochemical properties, plant nutrients, and root secondary metabolites and root depth and species. Among them, SAK shows a significant positive correlation with the physical and chemical properties of soil, and PC showed a significant positive correlation witg plant nutrition. Moreover, it can be seen that PC is very important. These are particularly significant for *G. glabra*. This indicates that when SAK and PC are sufficient, it is more conducive to the growth and development of *G. glabra*.

### 2.2. Diversity of Endophytic Bacterial Community

**Figure 2:**
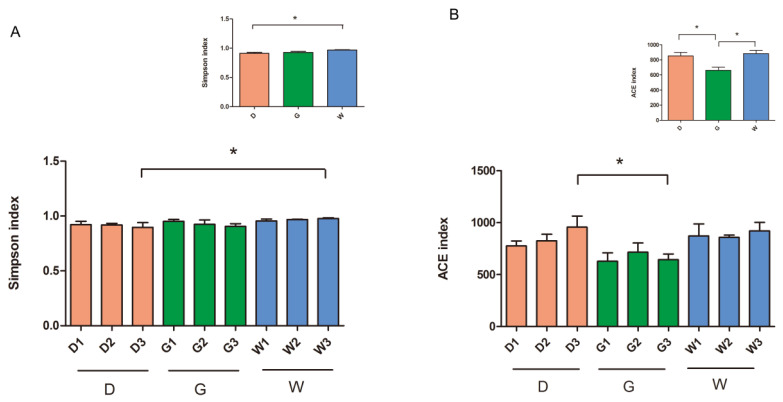
Alpha diversity analysis based on the Wilcoxon rank-sum test. (**A**) stands for the microbial diversity, (**B**) stands for species richness. Description: * represents a significant difference (*p* < 0.05) assessed by the Wilcoxon rank-sum test for analysis. The ordinate is the alpha diversity index (Simpson index and ACE index). The abscissa represents group names and is the same as that in Figure 1.

Wilcoxon rank-sum test results exhibit that the alpha diversity index (richness and diversity) of the endophytic bacterial community was significantly different among the three licorice samples (Figure 2), especially at a root depth of 40–60 cm (*p* < 0.05). Specifically, data analysis shows (Figure 2A) that the microbial diversity (Simpson index) of group W3 has a statistically significant increase compared to group D3. Meanwhile (Figure 2B), the species richness (ACE index) of group D3 is significantly better than that of group G3 (*p* < 0.05). For different varieties, W shows higher microbial diversity than group D, while group G is significantly lower than group D and group W in terms of species richness (ACE index) (*p* < 0.05). These results indicate that compared with the factor of root depth, different varieties of licorice have a more significant regulatory effect on the species diversity and community abundance of the symbiotic flora within the plants.

**Figure 3:**
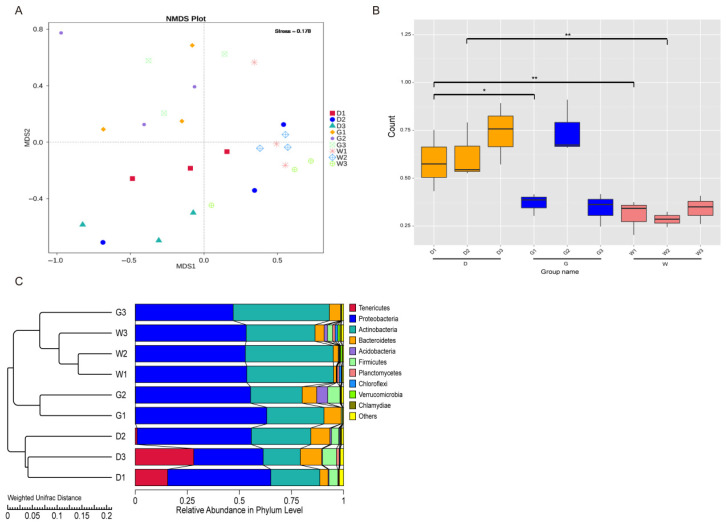
Beta diversity analysis of the endophytic bacterial community. Description: (**A**) Non-Metric Multi-Dimensional Scaling (NMDS) analysis, where each point in the diagram represents a sample, and samples from the same group are represented in the same color. The stress value (0.178) < 0.2 indicates that NMDS can accurately reflect the degree of difference between samples. (**B**) The significance test of beta diversity, where * and ** respectively represent the significant differences analyzed by the Wilcoxon rank-sum test (*p* < 0.05) and (*p* < 0.01). The abscissa represents group names and is the same as that in Figure 1. (**C**) Unweighted Pair Group Method with Arithmetic Mean (UPGMA) clustering tree based on the Weighted Unifrac distance, and the distribution of relative abundance of each sample at the phylum level.

The endophytic bacterial community was extracted and clustered by NMDS (Non-Metric Multi-Dimensional Scaling) analysis based on Bray–Curtis distance, demonstrating that root depth and species greatly influence the similarity of endophytic bacterial community structure (Figure 3A). The NMDS results are also validated by an unweighted Wilcox rank-sum test analysis based on weighted distance, which reveals substantial differences in endophytic bacterial communities’ Beta diversity (Figure 3B). There are particularly notable distinctions observed between D1 vs. G1 communities (*p* < 0.05), D1 vs. W1 populations (*p* < 0.01), and D2 vs. W2 microbiomes (*p* < 0.01). These results are consistent with the UPGMA (Unweighted Pair Group Method with Arithmetic Mean) results based on Weighted Unifrac suggesting that all the samples form three different clusters (Figure 3C), with each species grouped into a cluster. For example, D1, D2, and D3; W1, W2, and W3; and G1, and G2 indicate that the structure of the endophytic bacterial community in the root of medicinal licorice is significantly different among different species.

### 2.3. Differences in the Composition of Endophytic Bacterial Community

**Figure 4:**
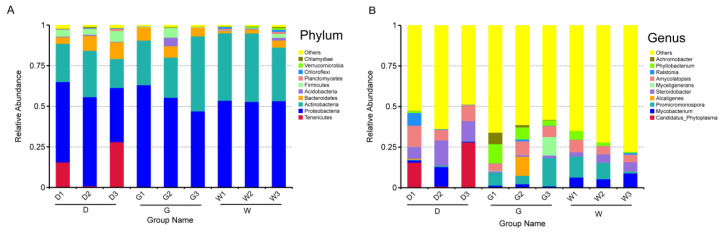
Histograms of relative abundance of the top 10 endophytic bacteria at the phylum (**A**) and genera (**B**) level of taxonomy. Description: the ordinate is the relative abundance of species, where “Others” means less or not annotated; the abscissa represents group names and is the same as that in Figure 1.

High-throughput sequencing of 16S rRNA amplicons identified 31 bacterial phyla across 50 classes, 115 orders, 231 families, 514 genera, and 274 species. Phylum-level analysis of the top 10 bacterial groups (Figure 4A) showed the following:

Proteobacteria dominance across samples (D1: 49.49%, D2: 54.85%, D3: 33.35%, G1: 63.02%, G2: 55.20%, W1: 53.51%, W2: 52.79%, and W3: 53.20%); and Actinobacteria as the secondary dominant phylum (D1: 3.560%, D2: 28.546%, D3: 17.875%, G1: 27.497%, G2: 24.769%, G3: 46.212%, W1: 41.613%, W2: 42.201%, and W3: 33.038%). In addition, Tenericutes was found to be the predominant phylum in D1 (15.574%) and D3 (28.053%). Proteobacteria and Planctomycetes decreased significantly in the G sample as the root depth decreased, while Bacteroidetes increased significantly in the D and W samples. The relative abundance of Acidobacteria, Firmicutes, Planctomycetes, Verrucomicrobia, and Chlamydiae increased significantly in the W sample.

In terms of genera (Figure 4B), *Candidatus Phytoplasma* occupied a large part of the relative abundance in D1 (15.564%) and D3 (28.037%), respectively. *Mycobacterium*, *Promicromonospora*, *Alcaligenes*, *Steroidobacter*, and *Myceligenerans* were found to be the predominant genera in the G3 (17.393%), D2 (15.545%), D2 (11.984%), G2 (11.687%), and G3 (11.422%) samples, respectively. Meanwhile, in sample D, the relative abundance of *Alcaligenes*, *Myceligenerans*, *Ralstonia*, and *Phyllobacterium* was significantly reduced with root depth. In the G sample, the relative abundance of *Steroidobacter* significantly increased with root depth, but that of *Phyllobacterium* and *Achromobacter* significantly decreased. In the W sample, *Promicromonospora*, *Myceligenerans*, *Amycolatopsis*, and *Phyllobacterium* abundance significantly reduced with root depth, but that of *Steroidobacter* and *Ralstonia* increased. In conclusion, the species and root depth of licorice significantly altered the relative abundance of the main dominant endophytic bacterial community composition (all values represent relative abundance percentages).

The composition information of dominant bacteria at each taxonomic level (class, order, family, and species) is enumerated in Supplementary Table S1. Specifically, Mollicutes, Gammaproteobacteria, Alphaproteobacteria, and unidentified_Actinobacteria dominate at the class taxonomic level; the dominant species at the order taxonomic level were unidentified_Mollicutes, unidentified_Gammaproteobacteria, Rhizobiales, Micrococcales, and Pseudonocardiales; the dominant species at the family taxonomic level were unidentified_Mollicutes, Burkholderiaceae, Promicromonosporaceae, Mycobacteriaceae, Rhizobiaceae, and Pseudonocardiaceae; the dominant species at the species taxonomic level were *Alcaligenes_faecalis*, *Ralstonia_solanacearum*, *Promicromonospora_umidemergens*, and *Streptomyces_ederensis*.

To examine statistics according to the Fresults of this study, from the perspective of licorice species, biomarkers with significant differences in abundance at the genus taxonomic level were found in the W sample (three taxa: *Streptomyces* and *Sphingomonas*) and G sample (three taxa: *Promicromonospora*, *Phyllobacterium*, and *Sinomicrobium*).

**Figure 5:**
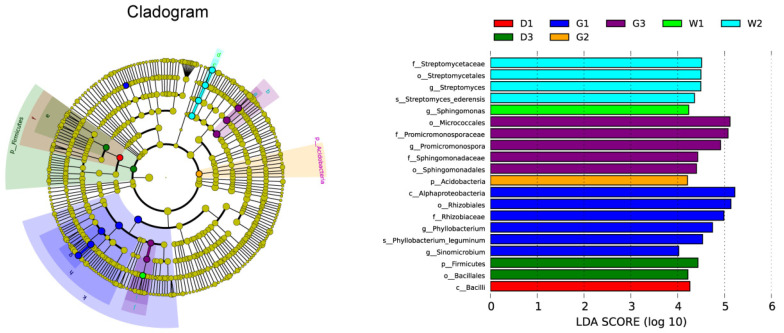
Linear discriminant analysis effect size (LEfSe) analysis of differences in endophytic bacterial community composition. Description: Cladograms (**left**) and LDA value distribution histogram (**right**) among three licorice species. In cladograms (**left**), the taxonomic level from phylum to species is from the inside out. The LDA value distribution histogram (**right**) figure shows the species with LDA scores greater than the set value (default setting is 4), that is, species with significant differences in different groups. The length of the histogram represents the influence of species with significant differences. The group name is the same as that in Figure 1.

To assess significant variations in endophytic microbial composition and host-specific associations, we conducted LEfSe (Linear Discriminant Analysis Effect Size) using nonparametric Kruskal–Wallis testing (Figure 5). In each group, a total of 20 biomarkers with significant differences were found in D1 (one taxon), D3 (two taxa), G1 (six taxa), G2 (one taxon), G3 (five taxa), W1 (one taxon), and W2 (four taxa).

### 2.4. Prediction of Endophytic Bacterial Community Function

**Figure 6:**
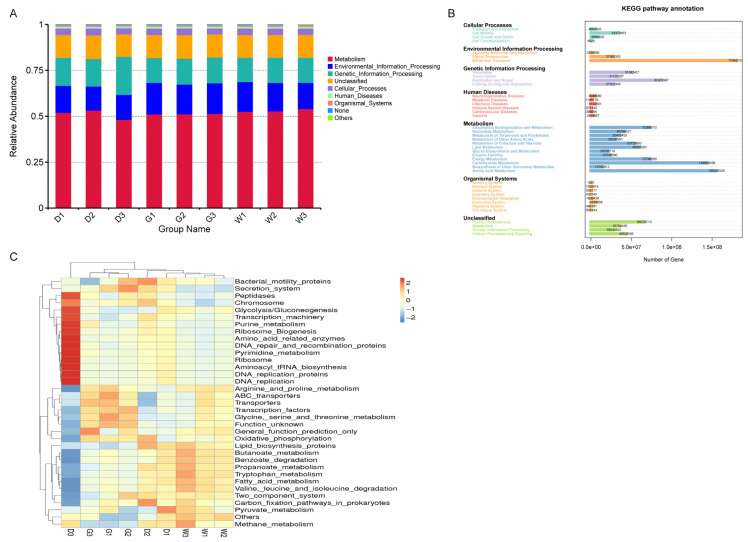
Prediction of endophytic bacterial community function. Description: (**A**) Histograms of relative abundance of the top 10 bacterial community functions at level 1. The ordinate is the relative abundance of bacterial community function, where “Others” means less or not annotated. (**B**) The number of genes at level 2, based on KEGG pathway annotation. (**C**) Cluster analysis based on the heatmap of endophytic bacterial community functions at level 3; the group name is the same as that in Figure 1.

KEGG (Kyoto Encyclopedia of Genes and Genomes) analysis annotated 5631 functional genes (KO), revealing 6 primary, 41 secondary, and 328 tertiary metabolic pathways. At level 1, metabolism (51.851%) was found to be the most dominant in each sample, followed by Environmental_Information_Processing (15.188%), Genetic_Information_Processing (14.847%), and Cellular_Processes (3.454%) (Figure 6A). Key metabolic subcategories included: xenobiotics biodegradation, amino acid/nucleotide metabolism, terpenoid/polyketide biosynthesis, and carbohydrate/lipid metabolism.

Level 2 analysis showed significant representation in membrane transport/signal transduction (Environmental_Information_Processing) and translation/replication (Genetic_Information_Processing) (Figure 6B).

At level 3, the functional information of the top 35 endophytic bacteria in terms of abundance was used to generate a clustering heat map (Figure 6C), which revealed that different licorice species had distinctly different dominant functional groups. In G samples, functional bacterium for Arginine_and_proline_metabolism, ABC_transporters, transporters, Transcription_factors, and Glycine_serine_and_threonine_metabolism were significantly dominant. In W samples, the functional bacterium for Lipid_biosynthesis_proteins, Butanoate_metabolism, Benzoate_degradation, Propanoate_metabolism, Tryptophan_metabolism, Fatty_acid_metabolism, and Valine, leucine_and_isoleucine_degradation were significantly dominant. There were 13 dominant functional groups in the D3 samples: Purine_metabolism and Pyrimidine_metabolism were related to Metabolism; and Chromosome, DNA_repair_and_recombination_proteins, and DNA_replication were related to Genetic_Information_Processing. From the above, it can be seen that the functions of the dominant endophytic bacterial communities within different licorice varieties also vary.

### 2.5. Relationship Between Plant and Soil Factors and Endophytic Bacterial Communities

**Figure 7:**
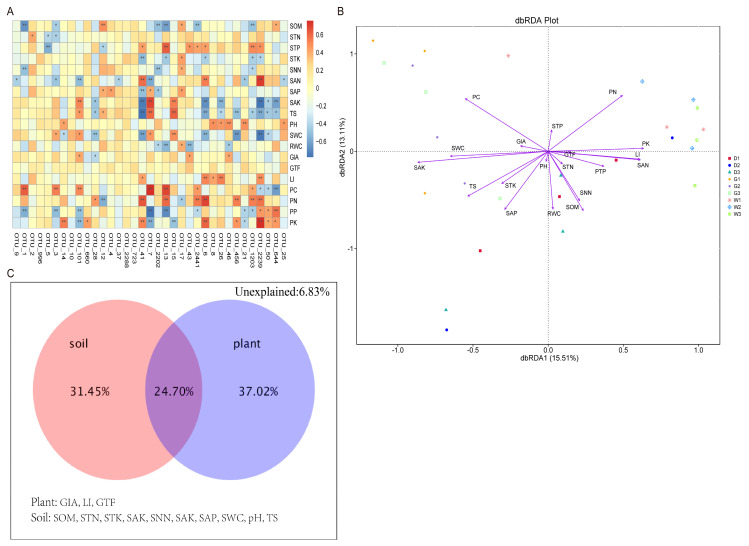
Relationship between root secondary metabolites, plant nutrients, and soil factors and microbial community. Description: (**A**) Heatmap of spearman correlation analysis between the top 35 OTUs, root secondary metabolites, plant nutrients, and soil factors. The mark * indicates significance test *p* < 0.05, and the mark ** indicates significance test *p* < 0.01; (**B**) Db-RDA analysis based on OTU levels, mainly used to reflect the relationship between microorganisms and environmental factors; (**C**) VPA between plant, soil, and endophytic bacterial community. Abbreviations are abiotic factors, which have identical meanings as described in Figure 1.

*Mycobacterium* OTU-6 and OTU-2239 (Actinobacteria) showed significant positive correlations with LI, PN, PK, SAN, and STP (R > 0, *p* < 0.05) but negative correlations with SAK, TS, STK, and SWC (R < 0, *p* < 0.05), similar to Streptomyces OTU-41 (Actinobacteria) (Figure 7A), which belonged to the genus *Streptomyces* of the Actinobacteria phylum.

Db-RDA (Bray–Curtis) identified key drivers of endophytic community distribution (28.62% of variance explained): (*r*^2^ = 0.593, *p* < 0.01), PN (*r*^2^ = 0.566, *p* < 0.01), PK (*r*^2^ = 0.396, *p* < 0.01), LI (*r*^2^ = 0.372, *p* < 0.01), SAK (*r*^2^ = 0.751, *p* < 0.01), SAP (*r*^2^ = 0.436, *p* < 0.01), SOM (*r*^2^ = 0.423, *p* < 0.01), SNN (*r*^2^ = 0.3, *p* < 0.05), SAN (*r*^2^ = 0.377, *p* < 0.01), TS (*r*^2^ = 0.489, *p* < 0.01), SWC (*r*^2^ = 0.41, *p* < 0.01), and RWC (*r*^2^ = 0.351, *p* < 0.01) (Figure 7B). Meanwhile, the results of VPA (variance partitioning canonical correspondence analysis) showed that plant factors (37.02%, plant nutrition and root secondary metabolites) were the most significant abiotic factors in the distribution of microbial communities, followed by soil factors (31.45%). Plant–soil interactions accounted for 24.70% of the differences in microbial community distribution; the unexplained part is the factor that has no significant impact on endophytic bacteria (Figure 7C).

**Figure 8:**
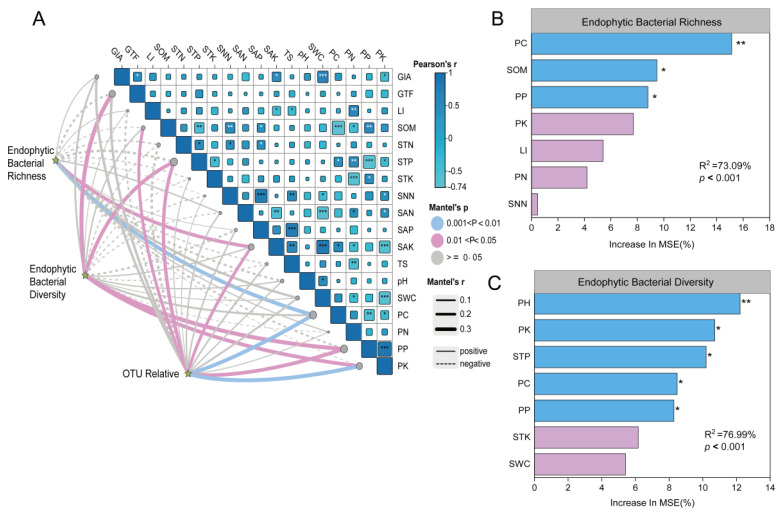
Mantel test analysis and random forest model predicted the vital factors influencing the root endophytic bacterial community of the Chinese medicinal herb Glycyrrhiza. Description: Mantel test analysis of endophytic bacterial community richness, diversity, and OTU relative abundance (**A**), and random forest model prediction of vital factors influencing endophytic bacterial community richness (**B**) and endophytic bacterial community diversity (**C**). ** means *p* < 0.01; * means *p* < 0.05.

As shown in Figure 8, the Mantel test results demonstrated significant correlations between the richness, diversity, and OTU relative abundance of the endophytic bacterial community and abiotic factors. Specifically, the richness of the endophytic bacterial community exhibited a significantly positive correlation with SAK and PC, the diversity of the endophytic bacterial community showed positive correlations with GTF, STP, PP and PK, and the OTU relative abundance of endophytic bacterial communities was positively associated with PC, PK, SOM, STP, SAK, and PP. The Spearman correlation analysis showed a significant positive correlation of SWC with GIA and SAK (*p* < 0.05); GIA’s positive association with soil water content suggests enhanced metabolite accumulation under more favorable moisture conditions. PK was positively correlated with PP (*p* < 0.05). TS was positively correlated with SAP and SAK (*p* < 0.05), which indicates that an increase in TS can lead to an increase in SAP and SAK in the soil. SAP were positively correlated with SNN (*p* < 0.05). At the same time, PK and SAN were significantly negatively correlated with SWC. PN was negatively correlated with STK. There was a significant negative correlation between PP and STP, which indicates that the nutrients required by licorice during its growth process are constantly absorbed from the soil. (Figure 8A). Meanwhile, the random forest model results identified key factors influencing the richness and diversity of the endophytic bacterial community. Specifically, PC, PP, and SOM were the key factors affecting the richness of the endophytic bacterial community (*p* < 0.001) (Figure 8B). Additionally, STP, PK, PH, and PC were determined to be the vital factors influencing the diversity of endophytic bacterial communities (*p* < 0.001) (Figure 8C).

## 3. Discussion

The investigation revealed significant variations in plant nutrients and secondary metabolites (GIA, GTF, and LI) among *G. uralensis*, *G. inflata*, and *G. glabra* roots (Table 1), aligning with Tao et al.’s findings [22]. Secondary metabolites are the byproducts of physiological processes that occur during plant cell differentiation and maturation as a result of coevolutionary forces between genes and the environment. On the one hand, most of the differences in secondary metabolite content in medicinal plants are due to differences in plant genotypes, such as variances in DNA sequences. Yang et al. [23] used genetic diversity analysis based on gene sequencing techniques to explain that psbA-trnH on chloroplast DNA sequences in three medicinal licorice species was significantly different between any two species.

Furthermore, GIA and LI, as the main secondary metabolites (8% of the total DW) in the rhizome of the licorice plant, protect plants against environmental stresses [24,25]. As a medicinal plant adapted to low soil fertility and drought conditions, the accumulation of secondary metabolites in roots is closely related to drought conditions. Plants have evolved a variety of mechanisms to respond to environmental stress, including pathways to increase secondary metabolite biosynthesis [26], where oxidative stress from reactive oxygen species (ROS) triggers enhanced biosynthesis [27,28]. Various studies have shown that secondary metabolites including GIA have antioxidant activity and can reduce the degree of oxidative damage to cells [29,30], with drought conditions upregulating triterpenoid saponin biosynthesis genes [31]. Therefore, drought-stressed plants increase the production of secondary metabolites to maintain the balance of their internal growth, which is consistent with our findings that the soil water content of *G. uralensis* is the lowest but the LI content in roots was the highest (Table 1). Interestingly, soil, water, and GIA content were the highest in *G. glabra*, suggesting that soil water content is not the only environmentally limiting factor for the production of secondary metabolites but is involved in a variety of complex interaction mechanisms.

From the Spearman correlation analysis in Figure 8, it can be seen that GIA’s positive association with soil water content suggests enhanced metabolite accumulation under more favorable moisture conditions. Plant nutrients need to be absorbed from the soil to change the accumulation of metabolites. It has been reported that the expression of genes related to the biosynthesis of plant secondary metabolites is affected by abiotic factors such as phosphorus availability [32]. Phosphorus nutrition can promote the biosynthesis of secondary metabolites, including terpenoids [33], as it stimulates terpenoids biosynthesis by increasing the accumulation of pyrophosphate compounds, including isopentene pyrophosphate (IPP) and dimethylallyl pyrophosphate (DMPP). These compounds are the most important precursors of terpenoid biosynthesis [34]. Similarly, Xie et al. [35] reported that increasing P contents could induce arbuscular mycorrhizal (AM) symbiosis and promote the accumulation of GIA and LI concentration in *G. uralensis*, implying the importance of increasing plant P uptake in AM symbiosis in regulating secondary metabolite biosynthesis. Nitrogen is generally considered to be one of the major limiting nutrients for plant growth. Biological nitrogen fixation, the biological activity responsible for converting molecular nitrogen to ammonia, is the most common source of nitrogen input in agricultural soils (even desert areas) and can help improve the fertility and productivity of low-nitrogen soils. Legume plants receive nitrogen by combining with rhizobia and generating a specialized organ on the host plant called a nodule [36]. Andrei, K.et al. [37] showed that rhizobium forms nodules in the root of licorice and promotes host nitrogen fixation through symbiosis in exchange for nutrients, thereby increasing the concentration of secondary metabolites in licorice plants.

The relationship between endophytes and their host plants is established by specific interactions and evolution, and it is crucial for the quality of secondary metabolites in medicinal plants [38]. In this study, HTS was used to identify the composition and diversity of endophytic bacterial communities, and results showed that the diversity of endophytic bacterial communities differed significantly between the three medicinal licorice species (Figure 2). These findings are consistent with the work of Gao et al. [39], which demonstrated that the types and quantity of endophytic bacteria were closely related to the genetic background of maize varieties. Meanwhile, beta diversity analysis revealed that the licorice species had a significant effect on the endophytic bacterial community structure when compared to root depth (Figure 3), implying that the endophytic bacterial community responded differently to the host plant genotype and ecological region (root depth). Numerous studies have revealed the isolation of some strains from host plants (with different genotypes) that can express parasitic or symbiotic specificity, depending on the host genotype that colonizes [40,41]. Host plants can recognize signals from microorganisms and respond to the colonization of bacterial communities, or vice versa. Thus, slight genetic differences in the endophytic and host plant genomes control the outcome of interactions. Some studies have shown that environmental heterogeneity determines the distribution range of host plants, and the fitness of endophytic bacteria largely depends on the fitness of host medicinal plants to the habitat [42]. Therefore, the colonization and dispersion of endophytic communities are largely determined by the host plants. On the other hand, according to our field investigation, the possibility of collecting three species of wild medicinal licorice in the same area is very small. Three different medicinal licorice species distributed in different areas, with specific differences between regions (ecological or environmental conditions, for instance, temperature, humidity, and soil nutrient levels), will indirectly or directly affect the endogenous bacterial population structure. Therefore, the population structure of three medicinal licorice endophytic bacterial communities from different sampling sites was significantly different, indicating that the community structure of endophytic bacterial communities may show some regional specificity, and endophytic bacterial communities in the same area showed a high degree of species taxonomic similarity. At some level, this is the result of environment–plant–microbial interactions and coevolution based on co-selective pressures.

The present study identified specific endophytic microbiomes within the roots of three given medicinal licorices and the relative abundance of endophytic bacterial community compositions correlated with host plants. For example, Proteobacteria was the dominant phylum in all samples, followed by Actinobacteria (Figure 4A), which was consistent with the findings reported by Lin et al. [43]. As the largest phylogenetic lineage with the most diversified phenotypes, Proteobacteria exists in many environments, including marine and grassland ecosystems [44]. Proteobacteria contains a variety of metabolic species and plays an important role in promoting plant growth and development and improving plant adaptation to the environment. *Mycobacterium*, *Promicromonospora*, and *Steroidobacter* were more abundant than other genera in this study (Figure 4B). These dominant bacteria can effectively produce and secrete plant growth hormone [45], The research by Ting Li et al. [46] also showed that the endophytic microbiota of wild licorice can effectively enhance the accumulation of secondary metabolites. Although the role of these dominant bacterial genera in the production of root exudates of medicinal licorice is still unclear, this information provides a direction for future research on improving the secondary metabolites of medicinal licorice.

Furthermore, our results showed that the relative abundance of Tenericutes and *Candidatus phytoplasma* was relatively high only in the samples of *G. inflata*, suggesting that the composition of the endophytic community is host-specific, evidenced in that endophytes limited to host plants usually have genetic characteristics and are described according to their hosts [11]. Studying this phenomenon can provide important information on the abundance of endophytic bacterial species and their ecological roles in medicinal plants. In addition, root depth can significantly change the relative abundance of the dominant phyla (Bacteroidetes, Firmicutes, Verrucomicrobia, and Planctomycetes) and genera (*Phyllobacterium*, *Achromobacter*, and *Promicromonospora*) in the root endophytic communities of three medicinal licorice species, also supported by LEFse analyses, which were based on the identification of different biological taxa (Figure 5). These results indicate that plant species and root depth are the main determinants of the relative abundance of endophytic community composition. We found some differences in the relative abundance of dominant species in plants and root depth (ecoregion) in our samples. The findings revealed that particular endophytic bacterial species preferentially grow in a specific ecological region and play different ecological roles than other endophytes in maintaining and modifying the structure and function of the medicinal licorice bacterial community [47]. These should be important considerations in the selection of endophytic bacteria for inoculum host plants to improve the quality and quality of licorice species in the future.

In this study, 5631 homologous functional genes (KO) were annotated by PICRUSt 2 software based on the KEGG database. By annotating the functions of endophytic bacteria at different levels, it was revealed that in levels 1 and 2, the functional genes related to Metabolism were always found to be significantly dominant, followed by Environmental-Information-Processing and Genetic-Information-Processing (Figure 6A). At level 2, the number of functional genes related to Membrane Transport, Amino Acid Metabolism, and Carbohydrate Metabolism was significantly larger (Figure 6B). Carbohydrate metabolism regulates the formation, decomposition, and interconversion of carbohydrates in organisms and therefore plays an important role in the whole biochemical process. Plants and microorganisms absorb ammonium salts, nitrates, and other inorganic nitrogen from the environment for the synthesis of proteins and nitrogen-containing substances via the process of amino acid metabolism [47]. A dry habitat will lead to the accumulation of a large number of substances regulating osmotic pressure in plant cells, in turn increasing the concentration of cytoplasm and reducing osmotic potential, which needs to be regulated by chemical signals transmitted through membrane transport [48]. The dominant functional genes enriched by the endophytic bacteria community were active in the rhizosphere of medicinal licorice, which may promote the adaptation of medicinal licorice to natural environments of drought and low-fertility soil and thus promote plant growth and development.

The significant changes in the taxonomy of numerous endophytic bacteria in the rhizosphere at different root depths of the three medicinal licorice resulted in significant changes in their metabolic activities. As expected, the KEGG metabolic pathway of the root endophytic bacterial community (i.e., Butanoate-metabolism, Propanoate-metabolism, Glycine-serine-and-threonine-metabolism, Tryptophan-metabolism, Fatty-acid-metabolism, Purine_metabolism, Pyrimidine-metabolism, and Arginine-and-proline-metabolism) was significantly altered in the three medicinal licorice species based on the metagenomic function of 16S rRNA in the root system (Figure 6C).

As a part of the host plant microbiome, the changes in endophytic bacterial community structure were closely related to the changes in plant growth conditions, plant nutrient elements, secondary metabolites, and soil pH and nutrient contents [49,50,51]. Our findings revealed that the three medicinal licorice plants had significant differences in soil physical and chemical properties (i.e., soil ammonium nitrogen, soil available potassium, soil organic matter, soil total phosphorus, soil nitrate nitrogen, soil total potassium, soil available phosphorus, and total salt) (Table 1), which were mainly related to the absorption and utilization of soil nutrients in their growth process and the participation of plant leaf drop in the process of the material cycle and energy flow in the soil ecosystem. Due to changes in the composition of different plant communities in ecological regions, the quality and quantity of plant litter deposited into soil subsystems may change significantly [52,53]. Previous studies have shown that changes in plant nutrients, soil physical and chemical properties, secondary metabolites, and resource access spatial patterns have significant effects on the rhizosphere endophytic bacterial community [54,55,56]. Consistent with these reports, our results showed that plant factors (i.e., PC, PN, PK, LI, and RWC) and soil physical and chemical properties (i.e., SAK, SAP, SOM, SNN, SAN, TS, and SWC) were important environmental driving factors for the distribution of endophytic bacterial communities (Figure 7B), which proves the existence of complex interactions between plants, endophytic bacterial communities, root secondary metabolites, and soil in medicinal plants, resulting in highly structured endophytic bacterial communities. Meanwhile, the VPA results supported the finding that plants factors (37.02%) were the most influential abiotic factors in the distribution differences among microbial communities, followed by soil factors (31.45%), with 24.70% accounted for by the interaction between plants and soil (Figure 7C), and 6.83% unexplained. Although the signaling mechanisms involved in the interactions (plant–endophyte, bacteria–soil) require further research to understand the mechanisms of action, our study provides a direction in the field to promote these beneficial multi-party interactions.

Through a Mantel test and random forest model prediction, the above analysis results can be verified, and the scope of key driving factors can be further narrowed. According to the results obtained, GTF, STP, PP and PK were positively correlated with endophytic bacterial community diversity, while SAK and PC were positively correlated with endophytic bacterial community richness (Figure 8A). Among them, PH (*p* < 0.01) (Figure 8B) and PC (*p* < 0.01) (Figure 8C) were the key factors associated with the diversity and richness of endophytic bacterial communities, respectively. This indicates that the richness and diversity of endophytic bacterial communities are mainly affected by changes in plants factors, which is consistent with the results in Figure 7C.

To sum up, soil parameter differences (Table 1) reflected species-specific nutrient cycling, influencing microbiome composition. Multivariate analysis identified plant nutrients (PC, PN, and PK) and soil properties (SAK and SAP) as major drivers (Figure 7B), with plant factors explaining 37.02% of community variation (Figure 7C). Mantel tests confirmed GTF, STP, and plant phosphorus (PP and PK) as diversity predictors, while pH and PC significantly affected richness (Figure 8A–C), emphasizing plant physiological impacts on microbiome assembly.

## 4. Materials and Methods

### 4.1. Sample Collection

Experiments were conducted in August and September 2019 in three natural distribution areas, Yiwu (43°33′58″ N, 94°81′86″ E), Hami City (42°84′48″ N, 93°54′80″ E), and Shihezi City (44°45′18″ N, 86°06′39″ E)), of medicinal licorice in northern Xinjiang, China. The three natural distribution regions have a temperate continental desert climate, and the soil type is sandy soil. The altitudes of Yiwu, Hami, and Shihezi are 1372.8 m, 806.1 m, and 340.2 m, respectively, while the average annual temperatures are 5.5 °C, 9.8 °C, and 8.1 °C. Annual rainfall is 105.8 mm, 33.8 mm, and 225 mm, respectively.

Field studies and sample collection were conducted in accordance with local institutional guidelines and legislation and did not involve protected species. To ensure representativeness of the experiments, a total of 9 plots (3 species * 3 plots) were sampled in this study. Homogeneous composite samples were collected from each plot. For each sample, according to the “Z” type [57], five *glycyrrhiza* plants with the same growth and no pests were randomly selected, and the roots were dug out as completely as possible. The plant roots were shaken repeatedly without damaging the root structure to separate the soil that was not firmly adhered to the roots, and rhizosphere soil samples (0–20 cm, 20–40 cm, and 40–60 cm, respectively) were obtained. Five soil samples were mixed to obtain a uniform composite soil sample, which was then placed into a self-sealing bag for soil physical and chemical analyses. Simultaneously, licorice roots were cut into pieces (0–20 cm, 20–40 cm, and 40–60 cm) using sterilized scissors. The root samples of each depth were divided into two subsamples. One part was placed in a sterile plastic bag for secondary metabolite analysis, while the other was placed in sterile bags, immediately transported to the laboratory in an icebox to remove microbial interference on the root surface, followed by sterilization according to a previous procedure [58], and then stored in liquid nitrogen for DNA extraction. At the same time, aboveground licorice plants were brought back to the laboratory in aseptic bags for nutrient analysis. A total of 63 experimental samples, including 9 aboveground plants, 27 soil samples, and 27 root samples (3 depths * 3 species * 3 replicates), were obtained for further processing.

### 4.2. Analysis of Soil Properties and Plant Nutrients

The rhizosphere soil samples and the aboveground parts of the licorice plant samples were air-dried to a constant weight in the laboratory. Before evaluating the physicochemical properties of soil and aboveground plant nutrients, the soil samples were passed through a 2 mm sieve to remove roots and debris. The aboveground part was crushed into a fine powder by a crusher. The pH of the soil was measured with a soil pH meter while using the conventional method (the ratio of soil to water was 1:5). Soil water content (SWC) and root water content (RWC) were measured by gravimetric analysis. Soil physicochemical characteristics and plant nutrients were determined according to Bao et al. [59] and our previously reported method [58]; total nitrogen (STN/PN) via sulfuric acid digestion (FOSS 1035 analyzer, Shanghai, China); total phosphorus content (STP/PP) by molybdenum antimony spectrophotometry; total potassium levels (STK/PK) using atomic absorption spectrometry; organic carbon (SOM/PC) by dichromate oxidation; nitrate/ammonium nitrogen (SNN/SAN) via calcium chloride extraction; available phosphorus (SAP) using sodium bicarbonate method; available potassium (SAK) by ammonium acetate extraction; and total salt (TS) through atomic absorption spectrometry.

### 4.3. Determination of Secondary Metabolites in Roots

Licorice root samples of each depth were placed in an oven (60 °C/72 h) and dried to a constant weight. The dried roots were crushed into a fine powder by a crusher. For secondary metabolite analysis, 0.2 g aliquots underwent ultrasonic extraction at room temperature (71% methanol, 250 W, 40 kHz), followed by centrifugation (12,000 rpm, 10 min) and filtration (0.22 μm) (SONICS, Newtown, CO, USA). HPLC (Agilent-1260 Infinity, Santa Clara, CA, USA) with an Agilent ZORBAX SB-C18 column (150 mm × 4.6 mm, 5 μm) was used to test for Glycyrrhizic acid (GIA) and Liquiritin (LI) [58]. Total flavonoid (GTF) content was determined using an ultraviolet spectrophotometer at a wavelength of 334 nm, while GIA (CAS#1405-86-3) and LI (CAS#551-15-5) standard substances were used as controls.

### 4.4. Microbial Community Analysis

Total genome DNA extraction utilized a commercial kit (Starvio Tiangen, Beijing, China), with quality verified by electrophoresis and spectrophotometry. The V4 region of 16S rRNA was amplified using specific primers (515F/806R) with PCR amplification.

Library preparation and (TruSeq^®^ DNA kit, Illumina, Waltham, MA USA) library quality control (Qubit^®^ 2.0 Fluorometer, Thermo Scientific (Waltham, MA, USA)/Agilent Bioanalyzer 2100 system) were conducted. After the library was qualified, it was sequenced using Illumina HiSeq2500 platforms, and 250 bp paired-end reads were generated at Beijing Compass Biotechnology Co., Ltd. (Beijing, China).

### 4.5. Biological and Statistical Analysis

Samples read were merged, and the raw tags were obtained using a very fast and accurate analysis tool, FLASH (V1.2.7, http://ccb.jhu.edu/software/FLASH/ accessed on 7 July 2023) [60]. QIIME (V1.9.1, http://qiime.org/index.html accessed on 22 May 2025) was used for filtering the original labels to generate high-quality labels [61,62]. The tag sequences obtained after the above treatment were compared with the Gold database by the UCHIME algorithm [63] to detect the presence of traces of removed chimeric sequences [64] so that the final effective tags could be obtained.

OTU (Operational Taxonomic Unit) clustering was performed using Uparse software (Uparse v7.0.1001, http://drive5.com/uparse/ accessed on 14 July 2023) [65] with a 97% sequence identity. The Silva Database (http://www.arb-silva.de/ accessed on 14 July 2023) [66] was used for OTU annotation to represent species sequence analysis (threshold set to 0.8–1). MUSCLE software (Version 3.8.31, http://www.drive5.com/muscle/ accessed on 14 July 2023) [67] was used for fast multi-sequence alignment. Then, the sample with the smallest amount of data was homogenized (53,053 reads for sample W.1.2). Finally, alpha and beta diversity analyses using the obtained data were carried out. All sequence data were deposited in the NCBI (PRJNA750271).

QIIME software (version 1.9.1) was used to calculate the observed species, Shannon, Simpson, Chao1, ACE, and Good coverage indices, displayed with R software (version 2.15.3). The Chao1, Shannon, and Good coverage indices can be used to evaluate the community richness, diversity, and sequencing depth of samples, respectively [68].

Based on the Kyoto Encyclopedia of Genes and Genomes (KEGG) database and the Phylogenetic Investigation of Communities by Reconstruction of Unobserved States (PICRUSt) bioinformatics program, the microbial community function was predicted using OTU gene information in 16S sequencing data [69]. Then, the pheatmap package was used for visualization. Distance-based redundancy analysis (db-RDA) and variance partitioning canonical correspondence analysis (VPA) were carried out with R soft (vegan data package) to explain the effects of environmental factors on the distribution of endophytic bacterial communities.

Both the Mantel test and random forest model prediction were implemented based on R language. The former relies on the devtools, linkET, and tidyverse packages. The latter relies on the randomForest, rfPermute, tidyverse, and A3 packages.

## 5. Conclusions

This study revealed the composition and structure of endophytic bacterial communities in the root systems of three medicinal licorice species at different root depths and explored their relationships with plant and soil factors. Specifically, the influence of licorice species on the composition of endophytic bacterial communities in the root system was significantly greater than that of root depth, and the community variation explained by species differences (37.02%) exceeded that of soil factors (31.45%). Among them, *Proteobacteria* and *Actinomycetes* are the dominant bacterial groups. PC and pH are the primary factors affecting community richness and diversity. SAK and PC are significantly positively correlated with community richness, while GTF, STP, PP, and PK drive diversity. Functional prediction shows that metabolism-related genes account for the highest proportion (51.85%), especially membrane transport and amino acid and carbohydrate metabolic pathways. Under drought stress, the accumulation of glycyrrhizin (LI) was the highest and positively correlated with the abundance of actinomycetes, indicating that endophytes may promote the synthesis of secondary metabolites by regulating stress responses. The above findings provide a theoretical basis for optimizing the quality of medicinal licorice by using specific bacterial communities and enhancing the growth and productivity of medicinal plants. Future research should focus on clarifying the molecular mechanisms of the interaction of endophytic bacteria in the plant–endophytic–soil cycle system, in order to utilize the potential of endophytic bacteria to enhance the quality of medicinal plants.

## Figures and Tables

**Table 1:** Differences in plant nutrients, root secondary metabolites, and soil physicochemical properties of three medicinal licorices.

	Variables	D	G	W
**Plant Nutrients**	PC (g/kg)	390.640 ± 10.396 b	441.021 ± 6.389 a	412.617 ± 2.304 ab
	PN (g/kg)	17.959 ± 0.323 b	21.021 ± 2.213 b	24.886 ± 1.110 a
	PP (g/kg)	1.460 ± 0.157 a	1.223 ± 0.015 a	1.274 ± 0.011 a
	PK (g/kg)	12.257 ± 2.296 a	9.021 ± 0.428 a	12.330 ± 0.488 a
**Root Secondary Metabolite**	GlA (%)	2.031 ± 0.091 b	2.751 ± 0.179 a	2.073 ± 0.267 ab
	GTF (%)	4.522 ± 0.103 a	4.582 ± 0.076 a	4.597 ± 0.050 a
	LI (%)	0.965 ± 0.110 b	0.941 ± 0.133 b	1.788 ± 0.319 a
	RWC (%)	56.756 ± 1.762 a	47.578 ± 1.165 b	48.563 ± 1.080 b
**Soil Physicochemical Properties**	SOM (g/kg)	27.990 ± 1.981 a	10.495 ± 0.817 b	14.744 ± 1.533 b
	STN (g/kg)	0.762 ± 0.121 a	0.693 ± 0.065 a	0.832 ± 0.084 a
	STP (g/kg)	0.537 ± 0.039 b	0.666 ± 0.012 a	0.712 ± 0.018 a
	STK (g/kg)	21.864 ± 0.441 a	20.771 ± 0.172 ab	19.743 ± 0.278 b
	SNN (mg/kg)	14.200 ± 2.443 a	3.552 ± 0.616 b	7.593 ± 2.387 ab
	SAN (mg/kg)	4.869 ± 0.382 b	3.326 ± 0.175 c	6.021 ± 0.288 a
	SAP (mg/kg)	9.678 ± 2.251 a	5.291 ± 0.841 ab	3.700 ± 0.963 b
	SAK (mg/kg)	180.031 ± 22.146 b	273.093 ± 19.963 a	81.207 ± 9.539 c
	TS (g/kg)	5.697 ± 1.716 a	4.894 ± 0.694 ab	1.033 ± 0.078 b
	PH	8.450 ± 0.068 a	8.831 ± 0.232 a	8.534 ± 0.067 a
	SWC (%)	4.923 ± 0.352 b	7.983 ± 0.638 a	3.583 ± 0.237 b

**Description:** Values are means ± standard errors; different lower-case letters represent a significant difference (*p* < 0.05), assessed by one-way analysis of variance followed by Bonferroni’s statistic test for multiple comparisons; the same letter indicates no significant difference (*p* > 0.05). D, G, and W: *Glycyrrhiza inflata*, *Glycyrrhiza glabra*, and *Glycyrrhiza uralensis*, respectively. Abbreviations: PC, plant carbon; PN, plant nitrogen; PP, plant phosphorus; PK, plant potassium; GIA, Glycyrrhizic acid; GTF, root total flavonoids; LI, Liquiritin; SOM, soil organic matter; STN, soil total nitrogen; STP, soil total phosphorus; STK, soil total potassium; SNN, soil nitrate nitrogen; SAN, soil ammonium nitrogen; SAP, soil available phosphorus; SAK, soil available potassium; TS, total salt; PH, soil pH; SWC, soil water content.

## Data Availability

The raw sequencing data used in this study were deposited in the NCBI Sequence Read Archive under BioProject accession number PRJNA750271. All data generated or analyzed during this study are included in this published article.

## Supplementary Materials

The following supporting information can be downloaded at: https://www.mdpi.com/article/10.3390/biology14070856/s1, Table S1: The composition of dominant bacteria at each classification level.
