# Supplementary material for: Analysis of Composition, Structure, and Driving Factors of Root-Associated Endophytic Bacterial Communities of the Chinese Medicinal Herb Glycyrrhiza
Source: Biology (Basel). 2025 Jul 15;14(7):856. doi: 10.3390/biology14070856 (PMC12292729; doi:10.3390/biology14070856)
Supplement: Supplementary file 1 [file biology-14-00856-s001.zip › biology-3686790-supplementary.pdf]

Analysis of composition, structure and driving factors of root-associated endophytic bacterial communities of the Chinese medicinal herb *Glycyrrhiza*

Zhilin Zhang [1], Aifang Ma[1], Tao Zhang[1], Li Zhuang[1] and Hanli Dang [1*]

[1] College of life Sciences, Shihezi University, Shihezi City, 832003, Xinjiang, China

[*] Correspondence and requests for materials should be addressed to Hanli Dang (E-mail: 1825368629@qq.com).

Supplementary information files

Supplementary table S1 the composition of dominant bacteria at each classification level

Taxonomy		D			G			W		
		1	2	3	1	2	3	1	2	3
Class	Mollicutes	15.574%	0.907%	28.053%	0.114%	0.170%	0.094%	0.053%	0.065%	0.065%
	Gammaproteobacteria	22.857%	22.568%	16.069%	17.551%	31.433%	8.249%	10.845%	9.895%	12.530%
	Alphaproteobacteria	23.206%	20.908%	10.885%	44.918%	22.671%	38.098%	41.371%	39.982%	35.757%
	unidentified_Actinobacteria	20.507%	25.316%	15.818%	24.304%	23.941%	43.850%	36.033%	37.170%	27.194%
	Deltaproteobacteria	3.414%	11.354%	6.375%	0.537%	1.079%	0.541%	1.283%	2.897%	4.890%
	Bacteroidia	3.590%	7.229%	9.721%	8.222%	6.942%	5.338%	1.187%	2.401%	4.318%
	Acidobacteriia	0.134%	0.067%	0.033%	0.005%	5.024%	0.006%	0.100%	0.068%	0.196%
	Thermoleophilia	1.067%	1.790%	0.608%	0.119%	0.211%	0.389%	4.520%	4.288%	4.384%

	Clostridia	0.491%	1.881%	3.146%	0.335%	3.813%	0.090%	0.065%	0.079%	1.178%
	Bacilli	3.707%	1.582%	3.546%	0.236%	2.023%	0.268%	0.128%	0.230%	1.178%
	Others	5.454%	6.399%	5.747%	3.660%	2.693%	3.077%	4.415%	2.927%	8.311%
	unidentified_Mollicutes	15.564%	0.892%	28.037%	0.112%	0.129%	0.093%	0.049%	0.064%	0.044%
	unidentified_Gammaproteobacteria	18.168%	19.798%	13.960%	9.266%	18.335%	3.503%	4.705%	7.635%	9.898%
	Rhizobiales	15.537%	12.312%	6.380%	33.965%	16.687%	22.246%	25.904%	22.092%	21.751%
	Micrococcales	0.676%	1.388%	0.390%	9.109%	5.785%	29.811%	14.165%	14.130%	4.848%
	Corynebacteriales	2.638%	12.077%	0.620%	1.358%	2.549%	1.423%	6.539%	5.579%	9.119%
**Older**	Pseudonocardiales	14.422%	6.708%	11.928%	6.447%	11.301%	9.247%	9.927%	5.682%	5.422%
	Myxococcales	1.822%	7.710%	2.741%	0.397%	0.293%	0.336%	1.007%	2.254%	3.057%
	Bacteroidales	1.071%	5.153%	7.592%	0.643%	1.860%	0.235%	0.047%	0.061%	2.643%
	Streptomycetales	0.078%	0.512%	0.197%	0.336%	0.257%	0.788%	1.700%	7.630%	4.339%
	Enterobacteriales	1.852%	0.359%	0.410%	0.331%	6.338%	0.774%	0.206%	0.198%	0.503%
	Others	28.171%	33.092%	27.747%	38.037%	36.467%	31.546%	35.752%	34.676%	38.376%
	unidentified_Mollicutes	15.564%	0.892%	28.037%	0.112%	0.129%	0.093%	0.049%	0.064%	0.044%
	Burkholderiaceae	9.399%	3.357%	0.890%	8.158%	16.225%	1.702%	0.834%	1.433%	2.430%
	Promicromonosporaceae	0.461%	0.879%	0.212%	8.361%	5.373%	28.815%	12.944%	10.149%	1.192%
**Family**	Mycobacteriaceae	1.340%	11.984%	0.380%	1.251%	2.134%	0.842%	6.325%	5.298%	8.857%
	Rhizobiaceae	5.619%	2.156%	1.474%	21.592%	12.033%	15.135%	12.864%	7.198%	3.871%
	unidentified_Gammaproteobacteria	7.559%	15.977%	12.635%	0.924%	1.282%	1.672%	3.394%	5.823%	6.466%
	Pseudonocardiaceae	14.422%	6.708%	11.928%	6.447%	11.301%	9.247%	9.927%	5.682%	5.422%

	Sandaracinaceae	1.405%	6.093%	1.898%	0.199%	0.106%	0.114%	0.312%	1.333%	1.758%
	Streptomycetaceae	0.078%	0.512%	0.197%	0.336%	0.257%	0.788%	1.700%	7.630%	4.339%
	Enterobacteriaceae	1.852%	0.359%	0.410%	0.331%	6.338%	0.774%	0.206%	0.198%	0.503%
	Others	42.301%	51.085%	41.941%	52.290%	44.822%	40.820%	51.444%	55.192%	65.119%
**Species**	*Alcaligenes_faecalis*	0.629%	0.030%	0.019%	0.040%	11.687%	0.031%	0.030%	0.028%	0.059%
	Promicromonospora_aerolata	0.299%	0.129%	0.108%	4.924%	4.614%	13.460%	0.869%	4.174%	0.331%
	Myceligenerans_halotolerans	0.070%	0.012%	0.010%	0.361%	0.133%	11.422%	0.027%	0.017%	0.025%
	Ralstonia_solanacearum	7.834%	0.364%	0.205%	0.117%	1.249%	0.231%	0.046%	0.070%	1.063%
	Promicromonospora_umidemergens	0.046%	0.684%	0.048%	1.773%	0.211%	3.587%	6.999%	2.688%	0.273%
	Streptomyces_ederensis	0.037%	0.068%	0.131%	0.025%	0.077%	0.023%	1.337%	5.222%	3.286%
	Phyllobacterium_leguminum	1.105%	0.061%	0.040%	6.934%	3.922%	2.328%	0.038%	0.035%	0.036%
	Neorhizobium_galegae	0.636%	0.184%	0.184%	0.097%	0.089%	0.630%	1.952%	0.454%	0.260%
	Nocardioides_albus	0.060%	0.043%	0.011%	2.165%	1.727%	0.249%	0.304%	0.667%	1.307%
	Bradyrhizobium_canariense	1.049%	1.430%	0.172%	0.030%	0.060%	0.093%	0.572%	1.157%	0.282%
	Others	88.236%	96.996%	99.073%	83.535%	76.233%	67.945%	87.827%	85.488%	93.078%

Description: Others: The sum of the undefined and unannotated parts. Group name: D, G and W: *Glycyrrhiza inflata*, *Glycyrrhiza glabra*, and *Glycyrrhiza uralensis*, respectively; 1, 2, and 3: root depth 0–20 cm, 20–40 cm, and 40–60 cm, respectively.
